# Point of Sale Advertising and Promotion of Cigarettes, Electronic Cigarettes, and Heated Tobacco Products in Warsaw, Poland—A Pilot Study

**DOI:** 10.3390/ijerph182413002

**Published:** 2021-12-09

**Authors:** Paweł Koczkodaj, Paloma Cuchi, Agata Ciuba, Elwira Gliwska, Armando Peruga

**Affiliations:** 1 Cancer Epidemiology and Primary Prevention Department, Maria Sklodowska-Curie National Research Institute of Oncology, 02-781 Warsaw, Poland; agata.ciuba@pib-nio.pl (A.C.); elwira.gliwska@pib-nio.pl (E.G.); 2 World Health Organization, Country Office for Poland, 02-326 Warsaw, Poland; cuchip@who.int; 3 Department of Social Medicine and Public Health, Doctoral School, Medical University of Warsaw, 02-007 Warsaw, Poland; 4 Department of Food Market and Consumer Research, Institute of Human Nutrition Sciences, Warsaw University of Life Sciences-SGGW, 02-776 Warsaw, Poland; 5 Tobacco Control Research Group, Epidemiology and Public Health Research Programme, Bellvitge Biomedical Research Institute-IDIBELL, 08908 Barcelona, Spain; perugaar@arperu.onmicrosoft.com; 6 Centro de Investigación Biomédica en Red de Enfermedades Respiratorias (CIBERES), 28029 Madrid, Spain; 7 Center for Epidemiology and Health Policies, Clínica Alemana School of Medicine, Universidad del Desarrollo, 7710162 Santiago, Chile

**Keywords:** tobacco, advertisement, promotion, youth, Poland

## Abstract

Prevalence of smoking and e-cigarette use among teenagers in Poland is high. Polish law bans most advertising and promotion for cigarettes, e-cigarettes, and heated tobacco products (HTPs). This study investigates marketing for these products at points of sale (POS) near secondary schools in Warsaw, Poland, noting if the advertising and promotion were allowed under current Polish laws. All POS within 250 m radii of five selected secondary schools in each of three Warsaw districts were assessed for tobacco and e-cigarette direct advertising, inside and outside; offers of gifts or promotional discounts; tobacco merchandising, and tobacco displays. Of the 112 POS surveyed, 83% exposed customers to some form of advertising or promotion of cigarettes, e-cigarettes, or HTPs; in 76%, advertising or promotion that violated Polish law was present. More than 80% of POS surveyed displayed tobacco products; in 19%, these products were displayed near products of interest to minors. POS density observed here was 30.3 per km^2^, higher than in other European cities. In Poland, a high proportion of POS near schools violates the law banning the advertisement and promotion of tobacco and nicotine consumer products through a dense tobacco retailer network.

## 1. Introduction

Smoking is still a critical public health challenge in Poland, particularly among youth. The latest available data from the Global Youth Tobacco Survey indicated that, in 2016, 15.6% of boys and 14.9% of girls aged 13–15 years old were current cigarette smokers in Poland. Of the 29 European countries with comparable data, Poland ranked in the top quartile in 2016 [1]. That same year, the prevalence of current electronic cigarette (e-cigarette) use was 28% among boys and 18.6% among girls 13–15 years of age. Teenagers in Poland were ranked as the highest users of e-cigarettes in Europe [2]. While the prevalence of current cigarette smoking has decreased slightly, although remaining high among teenagers since 1999 [3], the prevalence of e-cigarette use among Polish youth has increased significantly recently: from 6% in 2011 to 29.9% in 2014 [4]. To the best of our knowledge, there are no data on the prevalence of smoking heated tobacco products (HTPs) among youth in Poland.

Although the tobacco industry claims not to target the advertising and promotion of their products to young nonsmokers, scientific reviews of years of published research have concluded that marketing activities by the tobacco industry do directly influence the uptake of smoking by young people [5,6]. Many of the marketing techniques used by e-cigarette companies are similar to those used by the tobacco industry to promote conventional cigarettes [7].

Given the high prevalence of smoking and e-cigarette use among teenagers in Poland, this study explores the level and types of advertising and promotion of cigarettes, e-cigarettes, and HTPs present in Poland because teenagers are susceptible to these marketing techniques [8].

In Poland, the Protection of Public Health Against the Effects of Tobacco Use Act is a comprehensive ban on tobacco advertising, promotion, and sponsorship that applies equally to cigarettes, e-cigarettes, and HTPs [9]. This law bans advertising and promotion of cigarettes, e-cigarettes, and HTPs on TV and radio, in magazines and newspapers, on billboards and other outdoor media, and on the Internet, and also bans free distribution of products and promotional discounts (the ban came into force in 1999 and is universal, regardless of age) [10].

The Protection of Health Act also prohibits advertising and promotion of “tobacco products, tobacco accessories or imitations of tobacco products and accessories, and symbols related to tobacco use”. The definition of “tobacco products advertisement” includes “distribution of announcements, images of tobacco brands or symbols related to them, also: names and graphic symbols of tobacco product manufacturers … used to popularize the tobacco product brands”. The definition of “promotion of tobacco products” includes “public distribution of tobacco products … and other forms of encouragement to purchase or use tobacco products—with no exceptions for any means of reaching to a customer”. The Act also specifically prohibits displaying objects imitating tobacco product packaging at points of sale (POS). Collectively, the ban on tobacco advertising and promotion has been interpreted to include POS advertising and promotion [11].

Article 6(5) of the Act prohibits a “self-service system” of retail sale of tobacco products, except at duty-free stores. This is interpreted as prohibiting the placement of tobacco products within the direct reach of consumers, but not necessarily prohibiting product displays, and the courts in Poland have confirmed this interpretation. Therefore, product displays at retail POS, as they are understood in the WHO’s Guidelines for Implementation of Article 13 of the WHO Framework Convention on Tobacco Control (Tobacco advertising, promotion, and sponsorship), are not explicitly banned in the country.

Since other forms of tobacco advertising and promotion have been limited by law, the tobacco industry has relied more heavily on POS marketing techniques to promote smoking [12,13]. The presence of tobacco advertising and promotion at POS has been associated with tobacco smoking, particularly among youth. Children and adolescents who are more frequently exposed to POS tobacco promotion are 1.6 times more likely to have tried smoking and around 1.3 times more susceptible to future smoking than those less frequently exposed [14]. In Poland, 91% of youth aged 13–15 years of age reported visiting a POS during the last 30 days prior to completing the questionnaire [15].

There is evidence that compliance with the ban on advertising tobacco and related products in the mass media in Poland is high [16]. However, it is hard to discern if this is also true of POS advertising. Therefore, this pilot study was conducted to assess the presence of advertising and promotion of cigarettes, e-cigarettes, and HTPs in Warsaw, Poland, in POS located near to schools that children and youth are likely to visit to buy sweets, beverages, or food.

## 2. Methods

**Figure 1:**
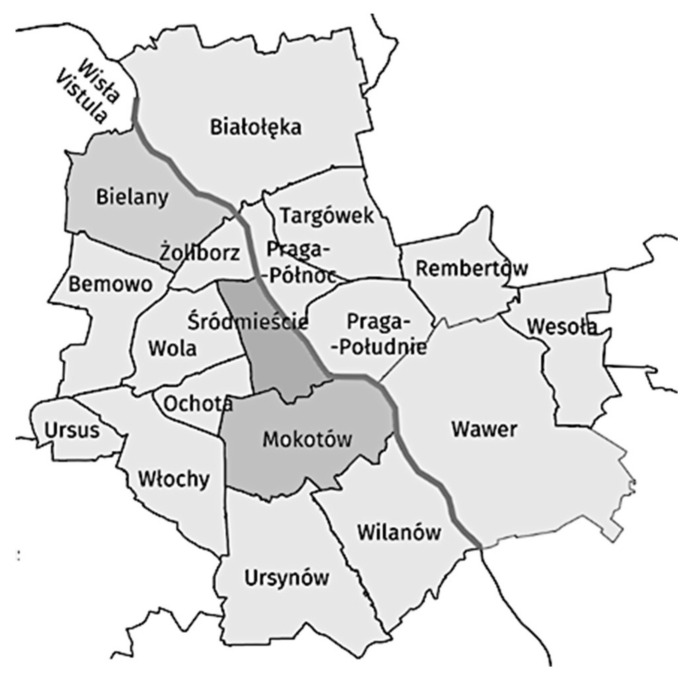
Map of the selected districts in Warsaw—Bielany, Śródmieście, and Mokotów.

A cross-sectional survey was carried out in all POS located in defined radii around a convenient sample of high schools in three districts of Warsaw, Poland. Three districts of Warsaw—Bielany, Mokotów, and Śródmieście—were selected to provide a range of aggregated area income levels (Figure 1). Bielany is a district with about 132,000 people; it has six high schools and has a population density, employment rate, and tax revenue below the Warsaw median. Mokotów has about 218,000 residents, and its population density, employment rate, and tax revenue approximate to the Warsaw median. Śródmieście has about 115,000 inhabitants, and has a population density, employment rate, and tax revenue above the Warsaw median. Mokotów and Śródmieście have 13 and 14 high schools, respectively [17].

In each district, five secondary schools were selected randomly from the list of high schools provided by the Education Office of the Capital City of Warsaw [18]. An area of about 250 m around each school was mapped out. Two authors (A.C. and E.G.) combed all the streets in the mapped areas on foot and identified and entered all potential POS open between 10 a.m. and 6 p.m.

A POS was defined as any venue where the products of interest could be sold to the public, independently of whether access to such venue was free or only by invitation or membership. POS included minimarkets and convenience stores, supermarkets, liquor stores, kiosks, and gas stations. Products of interest were combustible tobacco products, such as cigarettes; HTPs, such as IQOS and Glo; and e-cigarettes and their e-liquids.

**Figure 2:**
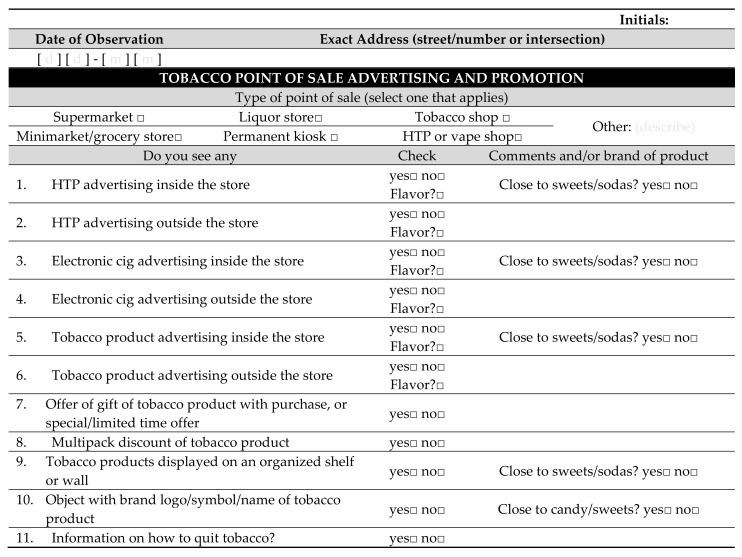
Standardized observation questionnaire used during the POS observations.

The field workers entered each identified POS that was open within the defined hours, incognito in pairs. A standardized observation instrument was used to record the presence or absence of different types of advertising and promotion for tobacco products (Figure 2). The field workers recorded the presence of advertising for e-cigarettes, HTPs, and tobacco products inside or outside the POS, and if flavored products were advertised. Also recorded were the presence of offers of tobacco product gifts with purchases, multipack discounts for tobacco products, the presence of objects branded with tobacco product logos, and the presence of specific display areas for tobacco products. In addition, the field workers noted if the adverts and product displays were situated less than 1 m away from products that might be attractive to children and young people, such as sweets and sodas. Other products attractive to children, such as comics or toys, are not typically found at the POS of products of interest for this study in Poland. Advertising and promotion types were defined according to the provisions of Polish law or the WHO Framework Convention on Tobacco Control by default.

The two fieldworkers completed the questionnaire for each POS by mutual agreement. The fieldworkers were allowed to work together for security reasons, and the observations cannot be considered to be truly independent. Therefore, inter-rater reliability was not calculated. The fieldworkers were in complete agreement with respect to the classification of advertising and promotion types; however, there were a few differences in observations between the fieldworkers with respect to which advertising was noticed. The fieldwork was conducted between 3 August and 23 December 2020. Because the study was carried out during the SARS-CoV-2 pandemic, not all the identified POS were open for observation.

In the analysis, the advertising and promotion of products of interest were considered in violation of the law if direct advertising happened inside or outside the venue or if objects branded with tobacco product logos were available, or if gifts or promotional discounts were offered with purchases of combustible products. The display of products of interest was not considered in violation of the law. STATA version 13 was used to calculate the proportion of POS showing the different forms of advertising and promotion under study. The protocol was not subject to ethical approval, as the observations did not involve human subjects, nor did retailers give prior informed consent to avoid revealing the presence of fieldworkers.

## 3. Results

A total of 123 POS were identified around the 15 surveyed schools, of which 112 were open for observation. Two of every three POS observed were minimarkets (66.1%). Kiosks were the second most common venue type, at 16% of the venues observed (Table 1).

Table 2 shows that 83% of the POS carried some form of tobacco or nicotine product advertising or promotion, and almost 76% did so in contravention of Polish law. None of the POS had any advertising outside; however, almost 45% of the POS had HTP adverts inside, and approximately one in five displayed adverts for cigarettes and e-cigarettes indoors. The proportions of POS surveyed that had indoor advertising of flavored cigarettes, HTPs, or e-cigarettes were 36%, 50%, and 67.3% of those that advertised these products, respectively (data not presented). Almost two-thirds of POS carried merchandising or objects with branding for cigarettes or other tobacco products, and about four in five displayed cigarettes prominently, one-fifth of them close to sweet and soda stands.

Table 3 reports the density of POS per square kilometer calculated as the ratio between the number of open POS surveyed and the total area surveyed in each district.

## 4. Discussion

In this study, 76% of POS located near to secondary schools in Warsaw exposed customers to various forms of advertising and promotion of cigarettes, e-cigarettes, and HTPs in contravention of Polish law.

The most common types of advertising and promotion were product displays and merchandising and objects bearing the logos and names of cigarette brands. While approximately 8 in 10 POS displayed products without contravening the law, almost 6 in 10 POS visibly carried objects branded with logos, such as change and counter mats, in violation of the law. When considering only direct advertising of cigarettes, e-cigarettes, and HTPs inside the POS through posters, banners, or video screens, about one in five of the POS observed advertised at least one of these products in contravention of the law. HTPs were the most frequently advertised form of tobacco at POS, despite their marginal consumption among the Polish adult population (0.4% in 2019) [19].

A 2019 study in Łódź, Poland, showed that all POS in the city advertised and promoted tobacco products and e-cigarettes in violation of Polish law, primarily through the presence of objects branded with logos but secondly through direct advertising [20]. This study and the results of our study suggest that the tobacco and nicotine industry is aggressively promoting its products, particularly HTPs, to youth at POS in Warsaw. Judging by the proportion of POS advertising flavored products, flavorings seem to be central to the marketing of e-cigarettes and HTPs, but not necessarily of cigarettes.

A 2019 study carried out by the National Institute of Public Health of Poland (NIPH) provides context to our study as it showed that POS are a significant source of exposure to advertising for teenagers. The study indicated that approximately 25%, 19%, and 15% of Polish students aged 15–18 years old recall having been exposed to cigarette, e-cigarette, and HTP advertising, respectively. Among those exposed to advertising, 34.6%, 18.9%, and 20.7% reported that the exposure to advertising for each of these products, respectively, happened at a POS [21]. For both e-cigarettes and HTPs, the advertising showed flavored products. Although our study did not measure the actual exposure of youth to advertising at POS, it did find that almost half of the observed POS advertised or promoted HTPs inside. This figure contrasts with the reported 20.7% of exposure at POS in the NIPH study. The higher figure in our study was possibly due to the increase in HTP marketing since the NIPH study was conducted. The market share by value of HTPs in Poland was estimated to be 5% of the total tobacco market at the end of 2020, with Poland being one of the markets with high potential in Europe for further fast sales development [22].

The findings of the “Report from a nationwide survey on attitudes toward tobacco smoking” prepared for the Chief Sanitary Inspectorate (CSI) in Poland in 2019 are aligned with our results on POS, providing a powerful method of exposure to advertising in Poland. The CSI found that about 15% of all smokers and 15% of nonsmokers were exposed to cigarette advertisements inside POS, and exposure at POS was the most frequent form of tobacco marketing [23].

The display of packaging of tobacco products itself is a form of advertising and promotion, and the law in Poland does not explicitly ban this. In our study, in almost 20% of the surveyed POS, the display of tobacco products was clear and easily noticeable by the investigators. In all cases, the display of products was located near products of interest to minors, such as sweets and sodas. The high visibility of tobacco products in POS are often visited by youth, and their placement near goods of interest to children is also, unfortunately, common in other countries. In Amsterdam, the Netherlands [24], 91.5% of 82 investigated POS had indoor visibility of tobacco products. In Scotland [25], 70% of 96 shops that were checked had tobacco products that were placed near goods of interest to children.

In this study in Poland, we found a high density of tobacco and nicotine retailers in these areas around secondary schools, although we cannot conclude that this density is different from areas that are not near schools. Retailer density not only increases the availability of tobacco products by increasing the opportunities to purchase these products but also enhances their visibility [26]. The existing evidence shows that a higher tobacco retail outlet density is associated with an increased prevalence of smoking behaviors among youth [27]. In our study, there was 30.3 open POS of tobacco per square kilometer near secondary schools. This is the highest concentration of POS near schools when compared with available data from cities in other European countries. Based on each country report, we estimated the retailer density by dividing the sum of POS detected in all school sampling areas by the sum of the surfaces of each sampling area estimated as the circle area corresponding to the reported radius around each school, usually of 250 meters: Slovenia (10.0 POS/km^2^) [28], Bosnia and Herzegovina (17.5 POS/km^2^) [29], Moldova (17.5 POS/km^2^) [30], Ukraine (18.4 POS/km^2^) [31], Romania (21.3 POS/km^2^) [32], Switzerland (25.3 POS/km^2^) [33], and Georgia (26.6 POS/km^2^) [34]. The percentage of POS presenting some form of tobacco advertisement is high in these countries, with levels of 72% in Romania, 81% in Ukraine, 92% Slovakia, and 95% in Georgia. The percentage in this study was 83%. Only Switzerland had a significantly lower percentage (39%) of POS presenting some form of tobacco advertisement [28,29,30,31,32,33,34].

We only found two POS that offered gifts or promotional discounts with the purchase of cigarettes. This may be an underestimate of the actual number of POS carrying such activities, given that these are promotional activities that may happen occasionally, and the visits of the fieldworkers during a short single time period may not have captured these promotions. The CSI study reported that 15% of smokers and 6% of nonsmokers were able to buy cigarettes at promotional prices, indicating that such illegal promotions are relatively frequent. In addition, the NIPH study indicated that 3.5%, 2.7%, and 4.5% of Polish students aged 15–18 years recalled cigarette, e-cigarette, and HTP promotions, respectively, through sales marketing and discounts, some of which occurred at the POS.

## 5. Conclusions

1. Several POS violate the law banning the advertisement and promotion of tobacco and nicotine consumer product in Poland. Efforts to enforce the law are suboptimal, and the governmental agencies responsible for enforcement should act swiftly.
2. The display of tobacco products at POS is prevalent and should be explicitly banned in Poland. Other countries have shown the way and the benefits of doing so [35]. A recent evaluation of the legislation banning tobacco displays at POS in Scotland showed multiple benefits. Among other benefits, the ban was associated with reducing the risk of smoking initiation in young people and the perceived accessibility of tobacco.
3. Further studies are needed on the advertisement and promotion strategies for nicotine products, particularly those addressed at young people. Field studies—similar to the one described in this article—can provide a real-time picture of the functioning of tobacco control laws, as well as any flaws and imperfections. The results will provide feedback for policymakers and stakeholders on what should be done in tobacco prevention, both in the short and long term. In our opinion, this pilot study should be continued in the future but in a broader form; for example, including rural areas, higher numbers of POS, and taking into account the identification of particular types of promotion and advertisement in the context of different variables such as types of POS and nicotine products.
4. A final recommendation to protect children and teens from the harms of tobacco is to reduce the high density of tobacco and nicotine retailers. Our study indicates that the density of tobacco POS in Warsaw may exceed the POS densities in other European cities. There are four primary policy approaches to reducing tobacco POS density: (a) prohibiting sales in specific retailer types; (b) prohibiting sales near youth-populated areas, including schools; (c) “declustering” POS by requiring them to be at a minimum distance from each other; and (d) capping the number of tobacco POS to a certain amount within a community. All these approaches effectively reduce retailer density reduction but outlawing the sale of tobacco products within a certain radius from schools tends to gather the most support [36].

## Figures and Tables

**Table 1:** Distribution of POS by type and availability for observation.

Type of POS	Closed		Open		Total	
	*n*	%	*n*	%	*n*	%
Gas station	0	0.0	2	1.8	2	1.6
Kiosk	6	54.5	18	16.1	24	19.5
Liquor store	2	18.2	3	2.7	5	4.1
Minimarket	2	18.2	74	66.1	76	61.8
Supermarket	0	0.0	10	8.9	10	8.1
HTP/vape shop	0	0.0	0	0.0	0	0.0
Other *	1	9.1	5	4.5	6	4.9
Total	11	100.0	112	100.0	123	100.0

* POS types hard to classify—their characteristics were closest to coffee shops or restaurants.

**Table 2:** POS with observed direct and indirect advertising by type of promotion.

Type of Advertising or Promotion at Each POS	*n*	% of Open POS *
Advertising of cigarettes—inside	25	22.3
Advertising of cigarettes—outside	0	0.0
Advertising of e-cigarettes or e-liquids—inside	22	19.6
Advertising of e-cigarettes or e-liquids—outside	0	0.0
Advertising of HTP devices or their inserts—inside	50	44.6
Advertising of HTP devices or their inserts—outside	0	0.0
Gifts or discounts with purchase of cigarettes and other tobacco products	2	1.8
Display of cigarettes and other tobacco products	91	81.2
Display of cigarettes and other tobacco products near sweets or soda	21	18.8
Merchandising and objects with cigarette and other tobacco product brands available	67	59.8
Any advertising or promotion	93	83.0
Any advertising or promotion law violation	85	75.9

* The denominator is the total of open POS observed since all POS are able to offer the full range of products of interest in this study and thus are able to advertise and promote them.

**Table 3:** Density of POS per km^2^ around schools by district.

District	School Areas Surveyed	Open POS in the Surveyed Area	Total Area Surveyed (km^2^)	POS Density (POS/km^2^) *
Bielany	5	17	0.41	41.5
Mokotów	5	43	1.92	22.4
Śródmieście	5	52	1.57	33.1
Total	15	118	3.9	30.3

* The density was calculated by dividing the number of open POS in the surveyed area by the total area surveyed in km^2^.

## Data Availability

Data available in a publicly accessible repository. The data presented in this study are openly available in FigShare at 10.6084/m9.figshare.17151089.
